# COVID-19 Vaccination Uptake and Myocarditis and/or Pericarditis Complications Among Chinese and South Asian Individuals in Ontario

**DOI:** 10.1016/j.cjco.2025.08.006

**Published:** 2025-08-22

**Authors:** Gordon Moe, Michael A. Campitelli, Milan Gupta, Chi-Ming Chow, Dennis T. Ko, Tomi Odugbemi, Isobel Sharpe, Peter P. Liu, Joseph Y. Chu

**Affiliations:** a Heart and Vascular Program, St. Michael’s Hospital, Department of Medicine, University of Toronto, Toronto, Ontario, Canada; b Department of Medicine, University of Toronto, Toronto, Ontario, Canada; c ICES, Toronto, Ontario, Canada; d Collaborative CME and Research Network, Brampton, Ontario, Canada; e University of Ottawa Heart Institute, University of Ottawa, Ottawa, Ontario Canada; f Schulich Heart Centre, Sunnybrook Hospital, Toronto, Ontario, Canada; g Division of Neurology, Toronto Western Hospital-University Health Network, Toronto, Ontario, Canada

**Keywords:** COVID-19 vaccination, Ontario, Chinese Canadian, South Asians

## Abstract

**Background:**

We previously demonstrated that the mortality rate following COVID-19 infection was higher in the Chinese population and lower in the South Asian population, compared to the general Ontario population. COVID-19 vaccines are effective in protecting against severe acute respiratory syndrome coronavirus-2 (SARS-CoV-2)-related diseases. Whether vaccination rates and the postvaccination risk of myocarditis and/or pericarditis are similar among ethnic groups is unclear.

**Methods:**

We conducted a population-based, retrospective cohort study using linked health datasets from Ontario to compare COVID-19 vaccination and postvaccination myocarditisand/or pericarditis rates among the Chinese, South Asian, and general populations. Populations were identified using a surname-based algorithm. The cohort was defined on December 1, 2020, coinciding with vaccine availability, and followed until June 30, 2022, for first and second dose. Myocarditis and/or pericarditis hospital admissions and emergency department visits within 42 days postvaccination were analyzed.

**Results:**

For the first dose, vaccination uptake was highest in the general population during the first 120 days. Afterwards, the Chinese and South Asian populations had a higher vaccine rate. A similar pattern was observed for the second dose. Postvaccination myocarditis and/or pericarditis rates were lower among the Chinese population (20 per 1,000,000 first doses) and the South Asian population (21 per 1,000,000 first doses), compared to that in the general population (51 per 1,000,000 first doses), with similar findings following the second dose. The standardized morbidity ratios, comparing observed vs expected myocarditis and/or pericarditis rates postvaccination were similar across the study groups.

**Conclusions:**

A time-dependent differential uptake and lower incidence of postvaccination myocarditis and/or pericarditis occurred among the Chinese and South Asian populations, compared to the general population. Our findings help inform the design of future research and health delivery programs.

## Résumé

**Contexte:**

Nous avons précédemment démontré que le taux de mortalité après l’infection par le virus de la COVID-19 était plus élevé chez les Chinois et plus faible chez les Asiatiques du Sud, comparativement à la population générale de l’Ontario. Les vaccins contre la COVID-19 confèrent une protection efficace contre les maladies à SRAS-CoV-2, mais on ignore si les taux de vaccination et le risque de myocardite/péricardite post-vaccination sont similaires entre les groupes ethniques.

**Méthodologie:**

Nous avons réalisé une étude de cohorte rétrospective populationnelle à partir d’ensembles de données de santé couplées de l’Ontario pour comparer les taux de vaccination contre la COVID-19 et les taux de myocardite/péricardite post-vaccination parmi les populations chinoise, asiatique du Sud et générale. Les populations ont été identifiées à l’aide d’un algorithme basé sur le nom de famille. La cohorte a été définie le 1^er^ décembre 2020 et coïncide avec la disponibilité du vaccin; elle a été suivie jusqu’au 30 juin 2022 pour la première et la deuxième dose. Les admissions à l’hôpital et les consultations à l’urgence pour une myocardite/péricardite dans les 42 jours suivant la vaccination ont été analysées.

**Résultats:**

Dans le cas de la première dose, le taux de vaccination a été le plus élevé dans la population générale au cours des 120 premiers jours. Par la suite, il a été plus élevé chez les Chinois et les Asiatiques du Sud. Une tendance similaire a été observée pour la deuxième dose. Les taux de myocardite/péricardite post-vaccinale ont été plus faibles chez les Chinois (20 cas pour 1 000 000 de premières doses) et chez les Asiatiques du Sud (21 cas pour 1 000 000 de premières doses), comparativement à la population générale (51 cas pour 1 000 000 de premières doses). Des résultats similaires ont été observés après la deuxième dose. Les rapports de morbidité standardisés comparant les taux observés et les taux attendus de myocardite/péricardite après la vaccination ont été similaires entre les groupes de l’étude.

**Conclusion:**

Un taux de vaccination différentiel en fonction du temps et une incidence plus faible de myocardite/péricardite post-vaccination ont été observés chez les Chinois et les Asiatiques du Sud, comparativement à la population générale. Nos résultats contribueront à orienter les travaux de recherche ultérieurs et les programmes de prestation de soins de santé.

Throughout the COVID-19 pandemic, race- and ethnicity-related differences in infection rates and adverse outcomes following infection have been described in multiple jurisdictions, but how these differences are influenced by systemic factors has not been reported.^1^ In Ontario, our previous work has demonstrated that the 30-day all-cause mortality rate was significantly higher for the Chinese population, compared to that of the general population, and it was lower in South Asians.^2^ In an extension study of this cohart in COVID-19 waves 1, 2 and 3, Chinese patients were found to have a higher 30-day mortality rate and more hospitalizations and emergency department visits. South Asians had a lower 30-day mortality rate but a higher incidence of hospitalization and emergency department visits. These outcomes were significantly different among waves 1, 2, and 3, with a greater mortality risk in all ethnicity groups for waves 2 and 3.^3^

COVID-19 vaccines are effective in protecting against severe acute respiratory syndrome coronavirus 2 (SARS-CoV-2)-related diseases in a real-world setting.^4^ Observational surveillance studies have shown an increased myocarditis risk following COVID-19 vaccination.^5^ A study conducted in Canada examined observed vs expected myocarditis rates and found that although absolute rates of myocarditis following COVID-19 vaccination were low, vaccine type, and patient age and sex were important factors to consider when assessing the risk of postvaccination myocarditis.^6^ However, whether COVID-19 vaccination rates, and the risk of postvaccination myocarditis, are similar across ethnic groups remains unclear.

## Objectives

The principal objectives of the current study were, therefore, to address the following unanswered questions:

1. What are the vaccination rates among the Ontario Chinese and South Asian populations, the 2 largest ethnic minority groups, compared to the rate in the general population?
2. What are the myocarditis and/or pericarditis rates among vaccinated individuals in the Ontario Chinese and South Asian populations, compared to the rate in the general population?

## Methods

### Study design, setting, and data

We conducted a population-based retrospective cohort study in Ontario to examine trends in COVID-19 vaccination uptake and cardiac complications following COVID-19 vaccination among the Chinese and South Asian populations, as compared to the general population. The Chinese and South Asian populations were chosen because they constitute the largest ethnic minority groups. Ontario is Canada’s most populous province (N = 14.2 million as of 2021), with an ethnically diverse and multicultural population. Through the Ontario Health Insurance Plan (OHIP), most residents are eligible to receive publicly funded healthcare services, including hospital and emergency department visits, as well as COVID-19 vaccines. Our study utilized Ontario healthcare administrative databases—which have been used extensively to research the COVID-19 pandemic—to define our study population, exposure groups, measures, and outcomes. These datasets were linked using unique encoded identifiers and analyzed at ICES (Toronto). ICES is an independent, nonprofit research institute; its legal status under Ontario’s health information privacy law allows it to collect and analyze healthcare and demographic data, without patient consent, for the purposes of healthcare system evaluation and improvement. The use of the data in this project is authorized under section 45 of Ontario’s Personal Health Information Protection Act (PHIPA) and was approved by the University of Toronto Research Ethics Board.

### Study population

Our study population consisted of all eligible individuals identified in the Ontario Registered Persons Database (RPDB) who were alive, were eligible to receive OHIP services, and were aged between 18 and 105 years as of December 1, 2020 (N = 12,416,916). This date was selected as our index date to coincide with the availability of COVID-19 vaccines. We excluded the following: those residing in areas with non-Ontario postal codes (N = 65,311); those with no contact with the healthcare system in the 5 years prior to the index date (N = 591,464); and those who were not continuously eligible for OHIP services in the year prior to the index date (N = 242,314). These exclusions were made to limit the study population to those who were likely residing within the province as of the study index date. To define the main exposure groups, we used a previously developed surname-based algorithm, applied to the Ontario RPDB, that classified residents as Chinese, South Asian, or in the general population, which includes all individuals not assigned to the Chinese or South Asian group and contains people across multiple racial and ethnic backgrounds.^7^ The list of surnames that was used has been validated and shows very high specificity ( > 99.5%), but low sensitivity (80% for Chinese; 50% for South Asian). Individuals who could not be assigned to an exposure group were excluded (N = 7979). Our final study population included 11,509,848 Ontario residents.

### Study measures and outcomes

The Ontario RPDB was used to capture basic demographic information (age, sex, and postal code) about all residents included in our study at the index date. A resident’s postal code was then linked to Statistics Canada data to define rural vs non-rural residence (living in a community with < 10,000 individuals) and neighborhood income quintile, an area-level income measure. We utilized the Canadian Institute for Health Information Discharge Abstract Database, the National Ambulatory Care Reporting System, and the OHIP databases to capture information on hospitalizations, emergency department visits, and physician claims, respectively. These interactions were used to identify individuals diagnosed with asthma, diabetes, hypertension, heart failure, and chronic obstructive pulmonary disease, utilizing validated algorithms,^8^, ^9^, ^10^, ^11^ and derive the Charlson Comorbidity Index based on data from the 5 years preceding the index date.^12^ The Ontario Drug Benefit Database and the Continuing Care Reporting System were used to identify individuals residing in long-term care (LTC) facilities.

Information on the dates and types of COVID-19 vaccines administered to our study population was ascertained using the provincial COVID-19 vaccine registry (COVAXON). We collected data on first- and second-dose COVID-19 vaccinations provided between the index date and June 30, 2022. Second-dose COVID-19 vaccinations administered < 21 days after the first dose were excluded.

We used the Canadian Institute for Health Information Discharge Abstract Database and the National Ambulatory Care Reporting System datasets to flag hospitalizations and emergency department visits for myocarditis and/or pericarditis (International Statistical Classification of Diseases and Related Health Problems, 10th revision, Canada [ICD-10-CA] codes: B33.2, I30.x, I32.x, I40.x, I41.x, and I51.4) occurring within 42 days of the first or second COVID-19 vaccination dose. We included hospitalizations and emergency department visits for which the diagnosis of an outcome of interest was suspected and/or included in any diagnostic code on the record. The diagnosis codes used to define myocarditis and pericarditis, as well as the decision to combine these events into a single outcome, were based on previous observational studies examining complications associated with the COVID-19 vaccine.^5^^,^^13^^,^^14^

### Statistical analyses

Standardized differences were used to compare baseline characteristics between the exposure groups, with a threshold > 0.1 representing a meaningful difference.^15^

To estimate the vaccination uptake in the cohort, Kaplan-Meier methods were used to estimate the time to first- and second-dose vaccination from the index date until the end of the study period (June 30, 2022), or until death, whichever occurred first. Crude and age- and sex-adjusted curves of 1 minus the Kaplan-Meier estimate (1-KM) were plotted for first- and second-dose vaccinations, stratified by LTC facility residence.^5^^,^^16^

We computed the crude and the age- and sex-standardized (using the 2021 Ontario population aged 18-105 years as the standard population) rates of myocarditis and/or pericarditis in the 42 days following the first- and second-dose COVID-19 vaccination. Multivariable logistic regression models were used to determine whether ethnicity was associated with outcome rates, after controlling for age, sex, and vaccine type. Separate models were run for the first- and second-dose vaccinations.

For each exposure group, we used yearly cohorts from 2015-2019 to determine the per-person-day rate of myocarditis and/or pericarditis among individuals aged 18-105 years during this baseline period before the onset of COVID-19. These baseline rates were stratified by age ( ≤ 19, 20-29, 30-39, 40-49, 50-59, 60-69, 70-79, ≥ 80 years) and sex. We estimated the number of expected cases by multiplying the per-person-day baseline rate of myocarditis and/or pericarditis with the 42-day risk period following first- and second-dose vaccination for each separate exposure group, age, and sex stratum. Within each exposure group, the expected number of myocarditis cases in each age and sex stratum was summed to determine the total number of expected cases following first- and second-dose COVID-19 vaccination. To compare the observed and expected numbers of cases for each exposure group, we calculated the standardized morbidity ratio (SMR) with 95% confidence intervals (CIs).^17^

## Results

Our final study population comprised 11,509,848 Ontario residents aged 18-105 years: 628,884 (5.5%), 471,668 (4.1%), and 10,409,296 (90.4%) were classified as being Chinese, South Asian, or part of the general population, respectively. Compared to those in the general population, Chinese individuals were less likely to be living in rural areas, and they had a lower prevalence of most chronic diseases, and lower Charlson Comorbidity Index scores, and South Asian individuals were younger, had a lower prevalence of living in the highest-income neighborhoods and in rural areas, and had a higher prevalence of diabetes (Table 1).

**Table 1:** Baseline characteristics for Ontario residents as of December 1, 2020, by exposure group

Characteristics	Chinese population (N = 628,884)	South Asian population (N = 471,668)	General population (N = 10,409,296)	StdDiff (Chinese vs general)	StdDiff (South Asian vs general)	StdDiff (Chinese vs South Asian)
Sex, female	336,874 (53.6)	233,418 (49.5)	5,340,221 (51.3)	0.05	0.04	0.08
Age, y, mean ± SD	48.39 ± 17.87	45.29 ± 17.30	49.38 ± 18.65	0.05	0.23	0.18
Age group, y						
≤ 19	14,596 (2.3)	12,697 (2.7)	283,893 (2.7)	0.03	0	0.02
20–29	96,533 (15.3)	83,927 (17.8)	1,605,403 (15.4)	0	0.06	0.07
30–39	115,445 (18.4)	110,378 (23.4)	1,734,374 (16.7)	0.04	0.17	0.12
40–49	103,584 (16.5)	88,852 (18.8)	1,637,092 (15.7)	0.02	0.08	0.06
50–59	123,451 (19.6)	69,631 (14.8)	1,839,287 (17.7)	0.05	0.08	0.13
60–69	90,723 (14.4)	53,337 (11.3)	1,635,202 (15.7)	0.04	0.13	0.09
70–79	52,472 (8.3)	36,350 (7.7)	1,062,056 (10.2)	0.06	0.09	0.02
≥ 80	32,080 (5.1)	16,496 (3.5)	611,989 (5.9)	0.03	0.11	0.08
Income quintile						
1 (lowest)	114,931 (18.3)	78,850 (16.7)	2,029,538 (19.5)	0.03	0.07	0.04
2	130,493 (20.7)	109,845 (23.3)	2,034,327 (19.5)	0.03	0.09	0.06
3	113,475 (18.0)	127,374 (27.0)	2,082,497 (20.0)	0.05	0.17	0.22
4	138,859 (22.1)	91,430 (19.4)	2,094,667 (20.1)	0.05	0.02	0.07
5 (highest)	129,248 (20.6)	63,250 (13.4)	2,138,667 (20.5)	0	0.19	0.19
Rural residence	4181 (0.7)	4142 (0.9)	1,184,736 (11.4)	0.46	0.45	0.02
Diabetes	71,661 (11.4)	88,467 (18.8)	1,291,934 (12.4)	0.03	0.18	0.21
Hypertension	137,280 (21.8)	120,503 (25.5)	2,851,566 (27.4)	0.13	0.04	0.09
Congestive heart failure	5904 (0.9)	7744 (1.6)	265,587 (2.6)	0.12	0.06	0.06
Asthma	57,737 (9.2)	66,825 (14.2)	1,697,113 (16.3)	0.21	0.06	0.16
COPD	24,489 (3.9)	14,944 (3.2)	876,129 (8.4)	0.19	0.23	0.04
Charlson Comorbidity Score						
0	608,350 (96.7)	449,776 (95.4)	9,729,388 (93.5)	0.15	0.08	0.07
1	7556 (1.2)	8739 (1.9)	283,818 (2.7)	0.11	0.06	0.05
≥ 2	12,978 (2.1)	13,153 (2.8)	396,090 (3.8)	0.1	0.06	0.05
LTC facility resident	2311 (0.4)	633 (0.1)	66,990 (0.6)	0.04	0.08	0.05

All data are presented as number (%), unless specified otherwise. COPD, chronic obstructive pulmonary disease; LTC, long-term care; SD, standard deviation; StdDiff, standardized difference.

A total of 10,063,466 individuals received at least one COVID-19 vaccine dose during follow-up (560,087 doses for the Chinese population; 431,504 doses for the South Asian population, and 9,071,875 doses for the general population). A total of 9,869,755 individuals received a second COVID-19 vaccine dose (552,745 doses for the Chinese population; 425,785 doses for the South Asian population, and 8,891,225 doses for the general population).

### Crude COVID-19 vaccination uptake rates

**Figure 1:**
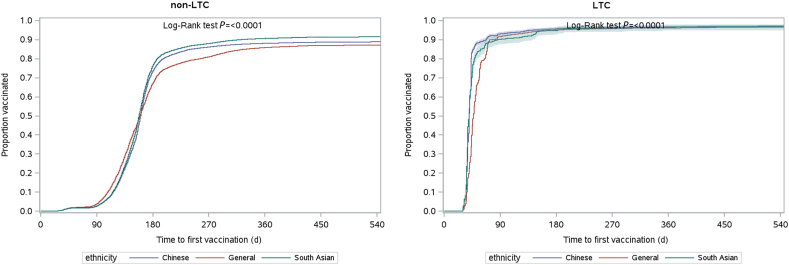
Time to first-dose vaccination by ethnicity until June 30, 2022 for the group not in a long-term care facility (non-LTC) and those in a long-term care facility (LTC).

The crude vaccination uptake rates for the first dose are shown in Figure 1. For individuals not in LTC, the rate of COVID-19 vaccination coverage was highest in the general population during the early part of the follow-up period. However, shortly after 120 days from the index date, the rate of vaccination coverage increased more rapidly in the Chinese and South Asian groups. By 180 days after the index date (around June 1, 2021)—although 67.2% of the general population group had received their first dose—the rate of vaccination coverage was significantly higher for the Chinese (73.6%, *P* < 0.001) and South Asian groups (76.6%, *P* < 0.001) vs that in the general population. From 180 days onward, the rate of vaccination coverage was consistently higher among the Chinese and South Asian populations. Trends in first-dose vaccination coverage among LTC facility residents are difficult to interpret. After 90 days following the index date (approximately March 1, 2021), more than 90% of residents in each group had received the vaccination. However, Chinese and South Asian LTC-facility residents received their first dose of the COVID-19 vaccine slightly earlier than did LTC-facility residents in the general population.

**Figure 2:**
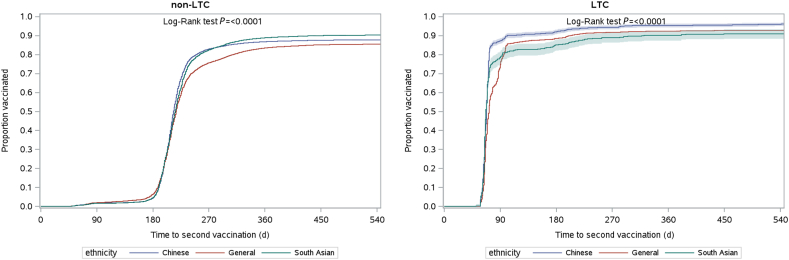
Time to second-dose vaccination by ethnicity until June 30, 2022 for the group not in a long-term care facility (non-LTC) and those in a long-term care facility (LTC).

Figure 2 contains plots for the second dose for individuals residing in and those residing outside of LTC facilities. Initially, the proportion of individuals who had received their second dose remained similar over time and across exposure groups. However, at around 240 days into the follow-up period (approximately August 1, 2021), differences in second-dose vaccination rates were observed—77.7% of the Chinese group (*P* < 0.001) and 75.7% of the South Asian group (*P* < 0.001) had received their second dose, compared to 69.5% of the general population. Thereafter, the rate of second-dose vaccination coverage was consistently higher among the Chinese and South Asian groups.

### Standardized COVID-19 vaccination uptake rates

**Figure 3:**
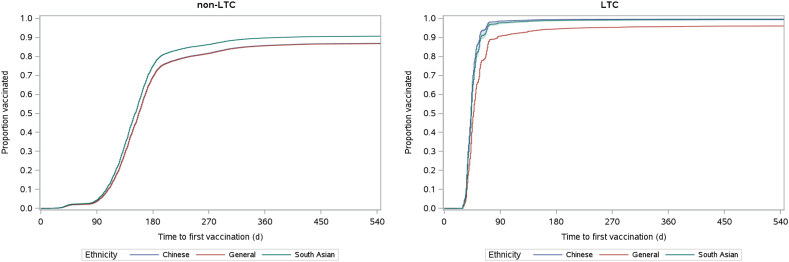
Age- and sex-adjusted time to first-dose vaccination by ethnicity until June 30, 2022 for the group not in a non-long-term care facility (non-LTC) and those in a long-term care facility (LTC).

Figure 3 displays plots adjusted by sex and age for the first dose of vaccination. For individuals not residing in an LTC facility, adjusting for age and sex removed the “crossover,”— that is, Chinese and South Asian groups transitioning from having lower to higher rates of first-dose vaccination coverage between 120 and 180 days after index, a pattern that was observed for the crude first-dose vaccination coverage. Given that the general population has a higher proportion of individuals aged 60-69 years, 70-79 years, and ≥ 80 years, and that the initial vaccine eligibility was tied closely to age, adjusting for age lowered the vaccination coverage estimates for this group during the early part of the follow-up period. After adjusting for age and sex, South Asians consistently had a higher first-dose vaccination uptake rate across the study period. In contrast, the Chinese and general populations had similar vaccination coverage over time. Adjusting the first-dose vaccination coverage estimates by age and sex did not impact the crude trends observed among LTC-facility residents.

**Figure 4:**
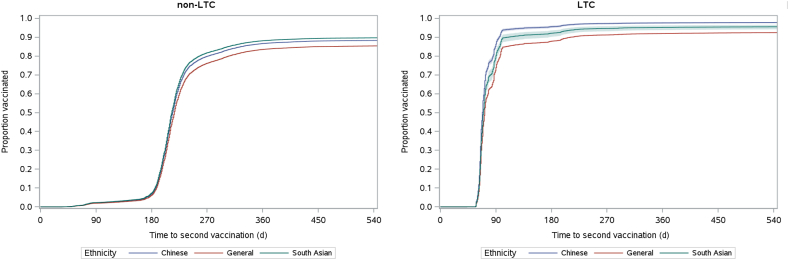
Age- and sex-adjusted time to second-dose vaccination by ethnicity until June 30, 2022 for the group not in a non-long-term care facility (non-LTC) and those in a long-term care facility (LTC).

Figure 4 displays plots for second-dose vaccination among those residing in and those residing outside LTC facilities at the index date, adjusted by sex and age category. For individuals not residing in an LTC facility, adjusting for age and sex had less of an impact on second-dose vaccination coverage, compared to first-dose estimates. Age was less of a driver of receipt of the second dose of vaccine, compared to receipt of the first dose. As in the crude analysis, the adjusted proportion of individuals who had received their second dose of COVID-19 vaccination remained consistent over time and across groups. Only after approximately 240 days into the follow-up period (approximately August 1, 2021) did differences in second-dose vaccination rates begin to emerge.

### Myocarditis and/or pericarditis rates following vaccination

The rates of myocarditis and/or pericarditis following the first and second dose of vaccination, overall, and stratified according to ethnicity, age, sex, and vaccine type, are shown in Tables 2 and 3, respectively. The crude rates of myocarditis and/or pericarditis in the 42 days following the first COVID-19 vaccination dose were lower in the Chinese group (19.6 cases per million doses) and the South Asian group (20.9 cases per million doses) vs the general population group (51.37 cases per million doses). After standardizing for age and sex, the rates of myocarditis and/or pericarditis following the first dose were similar to the crude rates for each group (19.75, 23.7, and 52.9 cases per million doses for individuals in the Chinese, South Asian, and general population groups, respectively). Overall, crude rates of myocarditis and/or pericarditis were higher in the 42 days following the second COVID-19 vaccination dose vs those in the 42 days following the first dose. However, lower rates were still found in the Chinese group (34.4 cases per million doses) and the South Asian group (30.5 cases per million doses) vs that in the general population group (65.7 cases per million doses) following the second dose. The age- and sex-standardized rates of myocarditis and/or pericarditis following the second COVID-19 vaccination dose were 35.3, 29.9, and 68.1 cases per million doses in the Chinese, South Asian, and general population groups, respectively. For both the first and second dose of COVID-19 vaccination, rates of myocarditis and/or pericarditis were numerically higher in male patients, those in the younger age categories ( ≤ 19 years, 20-39 years), and those receiving the Moderna vaccine.

**Table 2:** Crude rates of myocarditis and/or pericarditis in the 42 days following a first-dose vaccination for COVID-19, overall and by exposure group, age group, sex, and vaccine type

Study group	Number of myocarditis and/or pericarditis events	Number of COVID-19 vaccine doses administered	Rate per 1,000,000 doses (95% CI)
Overall	486	10,063,466	48.29 (44.09–52.78)
By exposure group (ethnicity)			
Chinese	11	560,087	19.64 (9.80–35.14)
South Asian	9	431,504	20.86 (9.54–39.59)
General population	466	9,071,875	51.37 (46.81–56.25)
By age group, y			
≤ 19	25	275,309	90.81 (58.77–134.05)
20–39	221	3,125,839	70.70 (61.69–80.66)
40–59	118	3,408,936	34.61 (28.65–41.45)
60–79	97	2,663,502	36.42 (29.53–44.43)
≥ 80	25	589,880	42.38 (27.43–62.56)
By sex			
Female individuals	169	5,223,853	32.35 (27.66–37.61)
Male individuals	317	4,839,613	65.50 (58.49–73.12)
By vaccine type∗			
Moderna	120	1,932,414	62.10 (51.49–74.25)
Pfizer	336	7,235,649	46.44 (41.60–51.68)

CI, confidence interval.

∗ Less common forms of the COVID-19 vaccine (eg, AstraZeneca, Covishield) were not included in table, to prevent the disclosure of cell sizes including ≤ 5 individuals in accordance with ICES contractual agreements with data providers.

**Table 3:** Crude rates of myocarditis and/or pericarditis in the 42 days following second-dose vaccination for COVID-19, overall and by exposure group, age group, sex, and vaccine type

Study group	Number of myocarditis and/or pericarditis events	Number of COVID-19 vaccine doses administered	Rate per 1,000,000 doses (95% CI)
Overall	616	9,869,755	62.41 (57.58–67.54)
By exposure group (ethnicity)			
Chinese	19	552,745	34.37 (20.70–53.68)
South Asian	13	425,785	30.53 (16.26–52.21)
General population	584	8,891,225	65.68 (60.46–71.23)
By age group, y			
≤ 19	62	268,083	231.27 (177.31–296.48)
20–39	284	3,042,884	93.33 (82.79–104.84)
40–59	130	3,353,864	38.76 (32.38–46.03)
60–79	104	2,631,635	39.52 (32.29–47.88)
≥ 80	36	573,289	62.80 (43.98–86.94)
By sex			
Female individual	192	5,134,093	37.40 (32.29–43.08)
Male individual	424	4,735,662	89.53 (81.21–98.48)
By vaccine type∗			
Moderna	324	3,397,345	95.37 (85.27–106.34)
Pfizer	285	6,223,450	45.79 (40.63–51.43)

CI, confidence interval.

∗ Less common forms of the COVID-19 vaccine (eg, AstraZeneca, Covishield) not included in table, to prevent the disclosure of cell sizes including ≤ 5 individuals in accordance with ICES contractual agreements with data providers.

When using a logistic regression model to examine the association between having myocarditis and/or pericarditis in the 42 days following the first dose and ethnicity, the odds of having myocarditis and/or pericarditis were significantly lower for individuals in the Chinese group (odds Ratio [OR] = 0.38, 95% CI = 0.21-0.70; *P* = 0.0016) and the South Asian group (OR = 0.41, 95% CI = 0.21-0.79; *P* = 0.0074) compared to those in the general population (Table 4). As with the first COVID-19 vaccination dose, the odds of having myocarditis and/or pericarditis within 42 days of the second dose were significantly lower in the Chinese group (OR = 0.52, 95% CI = 0.33-0.83; *P* = 0.0055) and the South Asian group (OR = 0.47, 95% CI = 0.27-0.81; *P* = 0.0063) vs the odds in the general population. For both the first and second doses, similar results were observed after adjusting for age group, sex, and vaccine type (Moderna, Pfizer, or others).

**Table 4:** Associations between exposure group and myocarditis and/or pericarditis in the 42 days following first- and second-dose vaccination for COVID-19

COVID-19 vaccination dose	Exposure group (ethnicity)	Unadjusted		Adjusted∗	
		OR (95% CI)	*P*	OR (95% CI)	*P*
First-dose vaccination					
	Chinese	0.38 (0.21–0.70)	0.0016	0.38 (0.21–0.70)	0.0017
	South Asian	0.41 (0.21–0.79)	0.0074	0.37 (0.19–0.72)	0.0034
	General	1.00 (reference)		1.00 (reference)	
Second-dose vaccination					
	Chinese	0.52 (0.33–0.83)	0.0055	0.55 (0.35–0.87)	0.0101
	South Asian	0.47 (0.27–0.81)	0.0063	0.43 (0.25–0.75)	0.0028
	General	1.00 (reference)		1.00 (reference)	

CI, confidence interval; OR, odds ratio.

∗ Models adjusted for age, sex, and vaccine type.

### Observed vs expected myocarditis and/or pericarditis rates

The rates of myocarditis and/or pericarditis in the 42 days following the first and second dose of COVID-19 vaccination were significantly lower in the Chinese and South Asian populations, compared to the rate in the general population. However, we also observed that the rates of myocarditis and/or pericarditis were significantly lower in the Chinese population (107.2 cases per million person-years) and the South Asian population (144.3 cases per million person-years) vs that in the general population (267.2 per million person-years) during a baseline period between 2015 and 2019, prior to the COVID-19 pandemic. Table 5 displays the SMRs calculated when comparing observed vs expected myocarditis and/or pericarditis cases in the 42 days following both the first and second doses of the COVID-19 vaccine. Given the small number of observed cases in the Chinese and South Asian groups, the CIs of our SMR estimates for these groups are wide, particularly for the first dose of the COVID-19 vaccine. Following the second COVID-19 vaccination dose, myocarditis and/or pericarditis cases increased among the Chinese population (SMR = 2.80; 95% CI = 1.69-4.36) and South Asian population (SMR = 1.84; 95% CI = 0.98-3.14) vs the expected incidence based on rates observed in 2015-2019. However, because the lower bound of the SMR CI for the South Asian population was 0.98, this increase was not considered statistically significant. Additionally, the general population also experienced a significant increase in myocarditis and/or pericarditis cases, compared to the expected incidence (SMR = 2.12; 95% CI = 1.95-2.30).

**Table 5:** Observed vs expected rates of myocarditis and/or pericarditis in the 42 days following first- and second-dose vaccination for COVID-19, by exposure group

Study population	First COVID-19 vaccination dose			Second COVID-19 vaccination dose		
	SMR	Lower 95% CI	Upper 95% CI	SMR	Lower 95% CI	Upper 95% CI
Chinese	1.60	0.80	2.84	2.80	1.69	4.36
South Asian	1.26	0.58	2.37	1.84	0.98	3.14
General	1.66	1.51	1.81	2.12	1.95	2.30

CI, confidence interval; SMR, standardized morbidity ratio.

## Discussion

A recent update states that the overall incidence of post-messenger (m)RNA vaccine myocarditis is low.^18^ Our current study is the first to examine the rates of both COVID-19 vaccine uptake and subsequent complications of myocarditis and/or pericarditis following vaccination among the 2 largest ethnic groups in Ontario, Canada, compared to those in the general population.

### Differential rates of COVID-19 vaccine uptake

Rates of first-dose COVID-19 vaccination uptake was initially highest in the general population. However, from 120 days onward, the Chinese and South Asian groups had a significantly higher vaccine uptake rate. The earlier receipt of the COVID-19 vaccine in the general population may be attributed to the fact that this population is older than the Chinese and South Asian populations, and therefore met age-based eligibility requirements sooner. A similar pattern was observed for the second dose of the COVID-19 vaccine.

Given the differences in uptake amongst ethnic groups, a national data study in England examined sociodemographic factors associated with vaccine uptake in adolescents.^19^ This study revealed large differences in vaccine uptake among ethnic groups, with those self-identified as having Chinese or Indian ethnicity having the highest vaccine rates, compared with the rate for those self-identified as Gypsy, Roma, or Black Caribbean. This pattern is consistent with our overall observation.

To the best of our knowledge, our study is the first to report a time-dependent relative COVID-19 vaccination rate among these 3 groups. The Chinese and South Asian groups had higher vaccination rates compared with that in the general population by day 120 of the follow-up period. The mechanisms underlying the time-dependent vaccination effect on the differential uptake between the Chinese and South Asian populations are unclear. In this regard, a previous study among adolescents in the United Kingdom has shown a higher vaccination rate of at least one dose in Chinese and Indian residents^19^; this finding is consistent with our observation on the second dose.

### Differential rates of post-vaccine myocarditis and/or pericarditis

The complication rate of post-vaccination myocarditis and/or pericarditis was lower among the Chinese and South Asian populations compared to that in the general population. Given the wide and overlapping nature of CIs from the SMR estimates of all 3 exposure groups, the definitive conclusion that Chinese and South Asian populations differed from the general population with respect to the numbers of observed vs expected myocarditis and/or pericarditis cases post-vaccination, when compared to historical trends, is not possible.

The numerically higher incidence from the Pfizer and Moderna vaccines, both mRNA-based preparations, is consistent with previous reports.^20^ A causal association between mRNA vaccines and myopericarditis has been suggested recently in a mouse model. Higher systemic levels of mRNA lipid nanoparticles due to inadvertent intravenous injection or rapid return from the lymphatic circulation were proposed to increase this risk.^21^

As stated earlier, a recent update states that the overall incidence of post-mRNA vaccine myocarditis is low.^18^ Therefore, a review of previous studies on the demographics of patients with vaccination-induced myocarditis is worthwhile. In a large Israeli healthcare system, among patients who had received at least one dose of the BNT162b2 mRNA vaccine, the estimated incidence of myocarditis was 2.13 cases per 100,000 persons; the highest incidence was among male patients aged between 16 and 29 years. Most cases of myocarditis were of mild or moderate in severity.^22^ A subsequent case series study of subjects aged ≥ 13 years vaccinated for COVID-19 in England between December 1, 2020 and December 15, 2021 also studied the association between vaccination and myocarditis, stratified by age and sex. Findings showed that the risk of myocarditis is higher after SARS-CoV-2 infection than it is after COVID-19 vaccination, and it remains modest after sequential doses, including a booster dose of BNT162b2 mRNA vaccine. The risk of myocarditis following vaccination is higher among younger men, particularly after a second dose of the mRNA-1273 vaccine.^23^

Regarding myocarditis complications, a recent study among Hong Kong adolescents examined the epidemiology of acute myocarditis and/or pericarditis following Pfizer Comirnaty vaccination between June 14, 2021 and September 4, 2021.^24^ The overall incidence of acute myocarditis and/or pericarditis was 18.5 per 100,000 vaccinated persons. The incidences after the first and second doses were 3.37 and 21.22 per 100,000 vaccinated persons, respectively. Among male adolescents, the incidences after the first and second doses were 5.57 and 37.32 per 100,000 vaccinated persons. In this Chinese population, a significant increase occurred in the risk of acute myocarditis and/or pericarditis among male adolescents, especially after the second dose of the Pfizer Comirnaty vaccine.

The mechanisms underlying the lower myocarditis rate following vaccination in the Chinese Canadians and South Asians are also unknown. One possible explanation is that these groups may seek out medical care less frequently. An interesting point to note is that ethnic diversity and immunogenicity among COVID-19 vaccines have been documented.^25^ One mechanism that is thought to produce variations in vaccine response is polymorphisms in major histocompatibility complex (MHC) genes, although polymorphisms in pattern recognition receptors (PRRs) or single-nucleotide polymorphisms (SNPs) also have been shown to be associated with variations in immunogenicity.^26^

### Limitations of the study

This study has potential limitations that warrant further discussion. First, our definition of myocarditis and/or pericarditis requires a detected visit to the hospital or emergency department, so we may be missing less-severe cases that presented elsewhere. Second, we used diagnosis codes for myocarditis and/or pericarditis that have unknown validity. However, these codes have been used extensively in other observational surveillance studies of complications following COVID-19 vaccination. Third, we relied on surnames as a proxy variable to identify those of Chinese and South Asian ethnicity, as population-based, self-reported ethnicity data were not available. The surname algorithm exhibits high specificity in identifying Chinese and South Asian individuals, but its sensitivity is somewhat limited. Surnames that are common to the Chinese or South Asian populations, but not unique to those groups, were deliberately excluded. Therefore, only a subset of these populations was identified, and our results may not be generalizable to the broader Chinese and South Asian communities in the province. Finally, social factors may be driving some of the ethnicity-related differences in COVID-19 vaccination rates and the rate of post-vaccination myocarditis and/or pericarditis observed in our study. Although an examination of these factors is out of the scope of this initial analysis, future work should attempt to understand them.

## Conclusions and implications of the current study

The observation of time-dependent differences in vaccine uptake among ethnic communities in Ontario suggests that ethno cultural, community-dependent, and policy factors can improve vaccine uptake. This finding may imply that ethnic community–tailored policy and practice, in collaboration with the community, can improve vaccination uptake. Research on the immunogenicity of candidate vaccines against COVID-19 is still in progress—especially on genetic and epigenetic contributions. However, the potential differences between ethnic groups are essential for future clinical trials to consider during study development, design, and post-study data analysis. Although the finding is reassuring that the overall incidence of complications such as myocarditis and/or pericarditis post-vaccination is very low, our data will enable health authorities to be informed regarding the potential effects of the vaccine on their local populations while providing guidance on which vaccines may be more appropriate for which specific patient cohorts.
